# Long-term disabilities after traumatic head injury (THI): a retrospective analysis from a large level-I trauma center in Saudi Arabia

**DOI:** 10.1186/s40621-017-0126-7

**Published:** 2017-11-01

**Authors:** Suliman Alghnam, Alaa AlSayyari, Ibrahim Albabtain, Bader Aldebasi, Mohamed Alkelya

**Affiliations:** 1 Population Health Section-King Abdullah International Medical Research Center (KAIMRC), King Saud Bin Abdulaziz University for Health Sciences (KSAU-HS), Riyadh, Saudi Arabia; 2 Department of Surgery-Hospital-NGHA, King Abdulaziz Medical City, Riyadh, Saudi Arabia; 3 Research Training and Development Section-King Abdullah International Medical Research Center (KAIMRC), Riyadh, Saudi Arabia; 4 Quality Management Section-King Abdullah International Medical Research Center (KAIMRC), King Saud Bin Abdulaziz University for Health Sciences (KSAU-HS), Riyadh, Saudi Arabia

**Keywords:** Traumatic head injury, Disabilities, Long-term outcomes, Motor-vehicle crash, ISS, RTS, Saudi Arabia

## Abstract

**Background:**

Traumatic head injuries (THI) are a critical public health problem worldwide, with more than 10 million individuals affected every year. In Saudi Arabia (SA), the burden of THI is unknown even though injury is the leading cause of death and a major cause of disability. Therefore, we aim to estimate the prevalence of long-term of disabilities among survivors of THI treated at a large level-I trauma center in Riyadh.

**Methods:**

The study included 258 patients, who were hospitalized due to a non-fatal THI between years 2005–2014. Patients (age = 16–60 years) were contacted via the phone and information about their Activity of Daily Living (ADL) and Instrumental Activity of Daily Living (IADL) was ascertained. Univariate analyses were performed to examine patients’ characteristics and to estimate the prevalence of any disability. Logistic regression was used to evaluate independent predictors of long-term disability.

**Results:**

Respondents were relatively young (mean age = 24.8; SD = 9.8), predominantly males (92.7%) and the majority sustained THI following traffic crashes (91.4%). The average time since the injury was 6.8 years (range = 3–12, SD = 2.6). Close to third of the sample (32.5%) reported at least some limitations in ADL or IADL. Regression analysis suggests that a one-unit increase in Revised Trauma Scale (RTS) was associated with 31% lower odds of disability adjusting for other covariates. While responders with a below high school education were 4.7 times more likely to report a disability than those with at least a college degree (*P* < 0.05).

**Conclusions:**

THI was associated with significant limitations in various aspects of everyday life. The magnitude and impact of THI in Saudi Arabia requires public health measures to prevent these injuries and to improve their health outcomes. Advocates may use these findings to educate the public about secondary and tertiary prevention and elicit support from policymakers to facilitate interventions toward reducing THI’s associated disabilities.

## Background

Traumatic head injury (THI) is a critical public health problem worldwide, with more than 10 million individuals affected every year (Hyder et al. 2007). THI kills over 50,000 annually (Center for Disease Control and Prevention 2017), mostly in developing countries (Abdullah et al. 2012; Shekhar et al. 2015). Today, 5 million individuals in the United States (US) and close to 7 million in Europe are living with a THI-related disability (Roozenbeek et al. 2013). In Saudi Arabia (SA), the burden of THI is unknown despite the fact that injury is the leading cause of death and a major cause of disability (Kassebaum et al. 2016). Every year, over 1.8 million individuals seek medical care following injuries, and injuries account for 17.8% of all deaths and 8.5% of emergency department (ED) visits (Ministry of Health 2015). The majority of injured patients are young adults (Alghnam et al. 2014), which may be associated with a significant loss in “years of productive life” (Schofield et al. 2016). This in turn may hinder the country’s progress and prosperity. In 2016, more than 40% of all reported disabilities were among those aged 10 to 24 (General Authority for Statistics 2016).

The term THI is used to describe any structural damage to the head, which may include structures other than the brain. In other literature, a more common term used is traumatic brain injuries (TBI), defined as an alteration in brain function, or other evidence of brain pathology, caused by an external force (Menon et al. 2010). Therefore, THI may include a wider spectrum of injury severity than TBI. Nevertheless, THI remains informative about the consequences posed by preventable injuries on population health.

The medical, psychological and socio-economic consequences of a THI have an extensive impact on individuals, families and communities. Aside from the physical impact, THI has wide-ranging effects on survivors, including behavioral, mental and social problems. Many studies based in developed countries have linked THI to depression (Underhill et al. 2003), sleep disturbances (Viola-Saltzman and Watson 2012), chronic pain (Nampiaparampil 2008), cognitive disorders (Arciniegas et al. 2002), and loss of communication skills (Dahlberg et al. 2006). Such impairments disrupt the ability to engage in daily activities within the home and community, which as a result impact Health-Related Quality of Life (HRQoL).

Over the past few decades, advances in trauma care have increased the number of THI survivors (Stein et al. 2010). The reduction in THI mortality has increased recognition of the importance of multi-dimensional concepts to describe health and disability, such as the International Classification of Functioning, Disability and Health (ICF). According to this model, functioning that is free from “disability” entails positive interactions between body function (i.e. psychological), body structure (i.e. anatomical), activities (i.e. mobility) and participations (World Health Organization 2001). Injuries, such THI, may disrupt these associations leading to impairment and disability.It is important to examine outcomes beyond hospital settings and investigate aspects that are associated with long-term disability, not only during acute care delivery. Therefore, exploring the functional outcomes associated with THI, is significant for establishing the long-term burden and consequences of THI.

The burden of long-term disabilities among THI survivors is poorly understood in SA. In the area of injuries, only two studies had examined outcomes other than mortality and found patients with spinal cord injuries to report low HRQoL (Al-Jadid et al. 2010, 2004) Unfortunately, no studies have addressed health outcomes of non-fatal THI. This may limit the ability to understand the magnitude of the problem and many policymakers continue to be unaware of THI’s burden and its significance to public health. As a result, THI prevention remains a neglected aspect of public health and governmental policies.

The public health approach emphasizes a community-based approach to injury prevention and improved collaboration from research to knowledge transfer to practice. Learning about the epidemiology and long-term outcomes of THI in healthcare settings can inform existing community-based programs of current challenges to boost prevention and facilitate support to increase investment in all types of prevention. Accordingly, the Saudi government has launched the *Saudi Vision 2030*, analogous to the *Healthy People 2020* program in the US (The Department of Health and Human Services 2010). One of the major goals of the *Saudi Vision 2030* has been to promote preventive care to reduce disease, disability and injury (Council of Economic and Development Affairs 2016). Faced with the considerations presented, this study aimed to assess long-term disabilities among THI survivors treated at a level-I trauma center. Findings from this study may provide basis for the prevalence of THI associated disability to facilitate prevention and to plan for the lifelong needs for patients to ultimately improve population health in SA.

## Methods

This retrospective cohort analysis used a trauma registry from King Abdulaziz Medical City (KAMC) in Riyadh, the capital of SA. In KAMC, patients who are eligible for treatment are only those working at the ministry of national guards, their parents, spouses and children. However, ineligible trauma patients can receive emergency care and can be admitted, if needed, until discharge or transfer to other hospitals. This 1500 bed hospital has over 200,000 visits to its ED annually, of which about 35% lead to hospital admission (Alkelya 2010). KAMC is one of the designated facilities in the middle east, according to the American College of Surgeon, that provides training in *Advanced Trauma Life Support* and meets the criteria of a level-I trauma center in the US (Tintinalli et al. 2010).

KAMC’s trauma registry includes hospitalized patients if they were admitted to the hospital following an acute injury or died at any point (including deaths upon arrival). In the ED, patients are triaged according to severity and urgency, and then admitted as needed and care is provided by respective specialty (i.e. surgery). A data registrar is designated to collect patients’ information and track their prognosis until hospital discharge. The data quality of the registry is evaluated annually by verifying collected items via medical records for 5% of the patients (Alhabdan et al. 2013).

### Patient population

One of the items on the checklist used to collect the registry’s data identifies the presence of head trauma. This is based on initial patient’s exam and confirmed by the CT scan report done for any patient with suspected head injuries. This include patients with head injuries listed as the primary or any additional diagnosis. All patients (16–60 years) with a record of THI, who were hospitalized for at least one night between January 2005 and December 2014 were eligible for inclusion. Patients who were declared dead either before or after hospital admission were excluded. Because information on post-discharge visits and outcomes are not included in the trauma registry, patients meeting the inclusion criteria were contacted via the phone to obtain information about their long-term outcomes. Three trained research coordinators ascertained the patient’s primary cellphone number from the medical record then contacted him or her to obtain informed consent. Once the patient agreed to participate, the coordinator collected demographics and measures of patients’ prognosis. The phone interview lasted no more than five minutes. For patients who are unconscious or unable to complete the information for the study, a proxy (family member) was asked to respond on the patient’s behalf. The research coordinators performed three attempts to contact each patient. If the patient did not answer, he or she was classified as a non-responder. The registry identified 1304 patients, but due to limited funding for data collection, a 50% of was randomly selected. No differences in demographic or clinical variables were observed between included and excluded patients (Appendix 1). Of the 652 included patients, two patients were deceased, 4 patients refused to be part of the study, 386 did not answer in any of the attempts to contact them, and 2 left the country. The final sample of the study comprised 258 patients (39.6% response rate).

### Independent variables

Several variables are collected in the registry including: age, gender, time of admission, mode of arrival, vital signs, injury mechanism, severity scores, procedures, length of hospital stay and discharge disposition (Alghnam et al. 2014). Severity scores include anatomic measures such as injury severity score (ISS) (Baker et al. 1974) and physiologic measures such as revised trauma scale (RTS) and Glasgow coma scale (GCS) (Lichtveld et al. 2008). These measures, which are collected in the ED, have been found to be associated with trauma outcomes (Kuhls et al. 2002).

### Dependent variable

The study evaluated two variables measuring functional status of daily activities. First, patients were asked about Activity of Daily Living (ADL), which focuses on patients’ ability to be independent. More specifically ADL asks whether the patient has difficulty in any of six main functions: bathing, eating, dressing, walking across a room, getting into or out of bed, and toileting (Khoei et al. 2013). The second outcome is Instrumental Activity of Daily Living (IADL). This outcome focuses on whether the patient needs supervision or help in activities such as using the phone, paying bills, or taking medications (Schootman and Jeffe 2003). Learning about these domains can facilitate understanding of the levels of assistance required for services related to caring for those with disabilities. The answer to the ADL or the IADL question is a three-level response: no difficulty, some difficulty, severe difficulty. Both ADL and IADL were administered in Arabic. These instruments have been validated in Arabic previously (Nasser and Doumit 2009).

### Statistical analysis

All analyses were performed using STATA 14 for Mac (STATA Corp., College Station, TX). Because both instruments contain three levels, they were dichotomized into two levels: no difficulty versus any difficulty (some or severe difficult). This was because the aim of the study is to examine the presence of any disability. Disability status was used as a binary variable (yes. vs. no) based on the occurrence of any reported limitation in either ADL or IADL. Descriptive statistics of the overall patient population and by disability status were calculated. Comparisons for categorical variables were performed using chi-2 tests and t-student tests for continuous variables. Significance level of *p* < 0.05 was declared as statistically significant.

Logistic regression analyses were conducted to evaluate potential significant predictors of long-term disability. The outcome was the presence of any disability. The following covariates were considered in the regression: mechanism of injury (motor vehicle-related injury, other), age in years categorized (16–25 as reference group, 26–45, 46 and older), gender, education at follow-up categorized (below high school, high school, college and above as the reference group), an indicator for transfer to surgery from the ED,ISS, and RTS (Lichtveld et al. 2008). Backward selection was used to identify independent predictors of long-term disability. Forward selection approach was also utilized and the finding was not changed. This study was reviewed and approved by the Institutional Review Board (IRB) at King Abdullah International Medical Research Center (KAIMRC).

## Results

Demographic and injury characteristics of disabled and non-disabled patients are shown in Table 1. The study population included 652 THI patients. Of which, 258 patients responded and were included in all analyses. The sample was predominantly males (92.6%) and young (mean age = 24.8 years, SD = 9.8). The finding also showed that the mean duration since the injury was 6.8 years (SD = 2.6) and most patients sustained injuries in motor vehicle crashes (91.4%), and were more likely to have a high school education or below (73.4%). Over 60% of the sample were admitted to the intensive care unit (ICU) during their stay and about a third had undergone surgery.

**Table 1:** Characteristics of the study population by disability status

Variable	Disabled	Non-disabled	All patients	*P*-value
	*N* = 84	*N* = 174	*N* = 258	
Age categorized [years] %				
16–25	53.57	71.26	65.50	<.01^a^
26–45	29.76	22.99	25.19	
≥46	16.67	5.75	9.30	
Male %	91.67	93.10	92.64	0.67^a^
Education %				
Below High school	34.94	10.40	18.36	<.01^a^
High school	46.99	58.96	55.08	
Above High school	18.07	30.64	26.56	
Mechanism of injury %				
MVC	92.86	90.80	91.47	0.58^a^
Others	7.14	9.20	8.53	
ICU %	67.86	56.32	60.08	0.07^a^
Surgery %	32.14	26.44	28.29	0.34^a^
Years since injury, (mean ± SD), median	(6.5 ± 2.7), 6	(6.9 ± 2.6), 6	(6.8 ± 2.6), 6	0.24^
RTS^b^, (mean ± SD), median	(9.9 ± 1.7), 10	(10.7 ± 1.3), 11	(10.4 ± 1.5), 11	<.01^^^
ISS^c^, (mean ± SD), median	(16.7 ± 9.3), 17	(13.3 ± 9.1), 12	(14.4 ± 9.3), 13	<.01^^^
Length of stay, median	40	15	22	

^a^Chi-2 test; ^ t-student test, ^b^RTS Triage-Revised Trauma Scale (higher indicates better physiological status), ^c^ISS Injury Severity Score (higher indicates worst injuries)

### Long-term disabilities

Based on the results, 32.5% of patients suffered long-term disabilities. Patients, who reported disabilities, were more likely to have lower levels of education (below high school: 34.9% vs. 10.4%, *P* < 0.01). Not surprisingly, they were also more likely to sustain severe injuries than non-disabled patients (ISS = 16.6 vs. 13.3; RTS = 9.9 vs. 10.7, both *P* < 0.01). Additionally, patients who reported any disability, were more likely to be admitted to the ICU and had longer hospital length of stay (LOS) compared to non-disabled patients.

Regression analysis of variables associated with disability identified RTS and education level as independent predictors. Despite lack of significance, age and gender were retained in the final model, as they are considered classical confounders Table 2. Regression analysis suggests that a one-unit increase in RTS was associated with 31% lower odds of disability adjusting for other covariates (OR = 0.69, 95% CI = 0.57–0.83). While patients with a below high school education were over four times more likely to report a disability than those with at least a college degree (OR = 4.7, 95% CI = 1.9–11.4). We compared responders and non-responders in several variables and found non-responders not to be different from responders in gender, ISS or RTS while non-responders were significantly older (mean age = 27 vs. 25, *P* < 0.01; Appendix 1) Table 2.

**Table 2:** Logistic regression analysis of risk factors associated with disability (N = 258)

Variables	Coefficent	Odds Ratio (OR)	Confidence Intervial (95% CI)
Age categorized [years]			
16–25	Reference	Reference	
26–45	.35	1.42	.72–2.79
46≥	.98	2.68	.96–7.46
Male	−.14	.86	.27–2.67
Education*			
College and above	Reference	Reference	
Below high school	1.54	4.70	1.93–11.41
High school	.22	1.25	.61–2.55
RTS*	−.36	0.69	.57–.83
Intercept	2.54	–	.57–.83

*P < 0.05

## Discussion

Our study found that about a third of THI patients, treated at a trauma center in Riyadh, suffer from long-term disabilities. This finding illustrates the significant burden of THI on the functional status of individuals in SA. As the country’s young population continues to age, the burden of THI disability will likely grow as the demand for ongoing medical care, rehabilitation and support expands. In addition, the increase in motor vehicle related injuries every year will add to the existing burden of THI disabilities (Alghnam et al. 2017). This underlines the importance of investing in primary prevention to reduce THI burden on population health and healthcare settings.

The burden found in our study is consistent with previous literature on the short-term implications of THI in SA (Al-Anazi 2004; Al-Habib et al. 2013; Chowdhury et al. 2014; WAHAB et al. 1989). Alhabdan et al. examined the epidemiology of THI in Riyadh and found it associated with increased mortality and healthcare utilization (Alhabdan et al. 2013). None of the previous Saudi literature, however, examined long-term functional outcomes, limiting the comparability of our results to others. Therefore, a major contribution of this study is setting a basis for estimates of long-term disability due to THI in order to advance knowledge by conducting further research and planning interventions to reduce its impact on population health.

The prevalence of long-term disability found in our study is similar to that of other countries like the US (Whiteneck et al. 2016) or China (Xu et al. 2011). However, a study by Selassie et al., using data from the state of South Carolina, found the incidence of disability to be 43% (Selassie et al. 2008). It is possible that this study captured more disabled patients than other studies because their disability definition was more comprehensive and thus, is likely to be sensitive to health deficits, which may explain this high estimate. The possibility remains that our study underestimated the prevalence of disability due differences in acute care between SA and developed countries. A previous study suggested that patients injured in motor vehicle crashes in SA were five times more likely to die in the hospital than patients treated in large US trauma centers (Alghnam et al. 2014). Suboptimal acute care may increase the risk of death among patients, who otherwise, would have survived had care been better. Therefore, survivors of severe THI may be more likely sustain long-term disability, which may change the overall prevalence of disability.

Another potential explanation of the discrepancy between our findings and others has to do with the definition of THI. KAMC’s trauma registry defines THI as any head injury documented during the initial examination by the treating physician and confirmed by CT scan. While other studies have used, for the most part, a more standardized definition, such as the Center for Disease Control and Prevention (CDC) case definition of TBI (Zaloshnja et al. 2008). This as a result reduces the comparability of patient populations as the former definition may include more mild injuries. Therefore, adopting international guidelines for defining TBI’s will improve the quality of the registry and facilitate comparability with other literature.

The study revealed that motor vehicle crashes were the underlying cause for most THI (91.4%). Whereas in the US, only 33.5% of THI leading to hospitalization are caused by motor vehicle crashes (Center for Disease Control and Prevention 2010) This is deeply troubling as SA continues to bear the toll of this substantial threat to population health. Investing in preventative strategies will likely facilitate a reduction in the number of individuals sustaining THI due to traffic crashes. For example, speed cameras and seatbelts may play important roles in reducing injury severity and mortality. A previous study in SA found speed cameras to be associated with 20% and 46% reduction in ISS and mortality respectively (Alghnam et al. 2017). Seatbelt is widely recognized as one the most effective and inexpensive public health interventions to reduce the burden of THI because it reduces the risk of ejection from the vehicle when crashes occur (Kwak et al. 2015). Yet, SA has a staggering low seatbelt compliance of around 5% while countries like the US has achieved 85% compliance (El Bcheraoui et al. 2015; Ibrahimova et al. 2011). Therefore, our findings highlight the need for further investment in road safety to increase seatbelt compliance through enforcement and education to reduce the incidence and consequences of THI. This is consistent with the goals of the National Transformation program (NTP), which is part of the *Saudi Vision 2030*. One the four goals the NTP in the health domain is reducing the burden of traffic injuries to improve population health (National Transformation Program 2016).

Our findings have several implications for public health policy and practice. First, the study findings highlight the need for further work in secondary and tertiary prevention. Disabled patients may require further treatment to improve their independence and ultimately, improve their quality of life. Second, we found those with low education levels to be more likely to report a disability independent of injury severity than those with high education, which concurs with previous studies (Schneider et al. 2014). It could be that those patients place a low value on follow up treatment and rehabilitation. In other word, health literacy of the importance of seeking rehabilitation could have played a role in this disparity of outcomes. Therefore, clinicians need to take this group into account, emphasize the importance of rehabilitation programs to increase compliance, and ensure their patients clearly understand the long-term goals of their treatment. Nevertheless, it is not unlikely that those with low education level had disabilities prior to sustaining THI or that educational attainment had been affected by sustaining THI, which may influence the estimates presented in this study. Third, our study found that two patients died after being discharged to their home. Several studies suggest that survivors of THI remain at risk of future mortality (Dams-O’Connor et al. 2015; Harrison-Felix et al. 2012). Therefore, further follow up of THI patients may be needed to improve their longevity following these injuries.

Future work should be done around barriers to receiving recommended rehabilitation services as this area of literature is lacking in SA. Even though unlike the US, healthcare services are free in SA, many patients may wait several weeks and perhaps months to receive care due to long waits. Consequently, this may hinder their ability to return to pre-injury health. Future studies should also utilize more comprehensive tools to evaluate self-reported health status, such as the Patient Reported Outcome Score (Patient-Reported Outcomes Measurement Information System 2017).

There are numerous limitations in this study that need to be acknowledged. First, the present finding is based on a repository from a single hospital and thus, generalizability to the population of Riyadh is unclear. Patients’ case mix may differ from the overall population as KAMC is known to be specialized in treating trauma patients, and therefore, more likely to receive severely injured patients. On the other hand, KAMC is considered as one of the best hospitals for trauma care nationwide. Thus, our study may underestimate the country’s THI disability because patients treated at other hospitals may be more likely to suffer THI disability. Second, although we would ideally use relative risk (RR) as the measure of association for this study design, the present study reported ORs which may overestimate measures of association. Third, the small sample size may have affected the power of the study. Therefore, future population-based studies are warranted to provide a more comprehensive view on the burden of THI nationwide. Fourth, our study suffered from a low response rate, which may introduce selection bias. Nevertheless, with the exception of age, our analysis did not reveal any difference in severity between responders and non-responders reducing the likelihood that they did not respond due to disability (Appendix 1). The possibility remains that because non-responders in our study were older, or for some other unknown confounder, they were more likely not to respond due to disability, which may bias our estimate. Another limitation has to do with the definition of THI described previously. This may lead to failure to identify all patients meeting the inclusion criteria. Therefore, using a standardized definition in the registry may improve future research. Finally, our estimates do not include THI treated in the ED and then released, or those transferred to other hospitals. This as a result may underestimate the burden of THI.

Nevertheless, the study has several strengths. First, it is the first attempt to estimate the overall burden of permanent disability among patients suffering from non-fatal THI in SA. Second, the study ascertained health outcomes of patients injured many years ago, which allowed capturing long-term disabilities. Finally, the use of multidimensional constructs to examine disability after THI provides a more comprehensive description of the burden of injuries.

## Conclusions

In summary, we found that about a third of patients, who were treated for THI, suffer a permanent disability. The burden of THI requires investment secondary and tertiary prevention to improve health outcomes. In addition, there is a paucity of injury prevention programs in SA and these findings are a testament to the need for such initiatives, especially in the area of motor vehicle crashes. Advocates may use these findings to educate the public about prevention and elicit support from policymakers to facilitate prevention and improve population health.

## Appendix 1

**Table 3:** Sensitivity analysis comparing the study population to the excluded population

Variable	Study population	Excluded sample	*P*-value
	*N* = 652	N = 652	
Gender %	92.6	92.7	0.91^a^
Age, mean	26.9	26.8	0.76^
Length of stay, mean	51.5	49.2	0.56^^^
Revised trauma scale,mean	10.4	10.3	0.26^
Injury severity score, mean	13.9	14.4	0.33^
Intensive care unit admission %	62.7	60.8	0.44^a^
Surgey %	28.3	26.4	0.41^a^

^a^Chi-2; ^T-test

**Table 4:** Sensitivity analysis comparing responders and non-responders on several variables

Variable	Responders	Non-responders	Total	*P*-value
Gender %	92.6	93.9	93.4	0.52^a^
Age, mean	25.4	27.9	26.9	<0.01^
Length of stay, mean	48.7	54.4	52.5	0.42^^^
Revised trauma scale, mean	10.4	10.4	10.4	0.88^
Injury severity score, mean	14.4	13.7	10.0	0.33^
Intensive care unit admission%	60.0	61.1	60.7	0.78^a^
Surgery %	28.2	25.6	26.7	0.45^a^

^a^Chi-2; ^T-test

## Abbreviations

ADL: Activity of Daily Living
CDC: Center for Disease Control and Prevention
ED: Emergency Department
GCS: Glasgow Coma Scale
HRQoL: Health-Related Quality of Life
IADL: Instrumental Activity of Daily Living
IRB: Institutional Review Board
ISS: Injury Severity Score
RTS: Revised Trauma Scale
SA: Saudi Arabia
THI: Traumatic head injury
US: United States
